# Continuous real time 3D motion reproduction using dynamic MRI and precomputed 4DCT deformation fields

**DOI:** 10.1002/acm2.12953

**Published:** 2020-07-02

**Authors:** Damien Dasnoy‐Sumell, Kevin Souris, G. Van Ooteghem, Benoit Macq

**Affiliations:** ^1^ Institute of Information and Communication Technologies Electronics and Applied Mathematics Universite Catholique de Louvain Louvain‐la‐Neuve Belgium; ^2^ Institut de Recherche Experimentale et Clinique (IREC) Molecular Imaging, Radiotherapy and Oncology (MIRO) Universite Catholique de Louvain Brussels Belgium

**Keywords:** IGRT, image deformation, motion model, MRI, real‐time tracking

## Abstract

Radiotherapy of mobile tumors requires specific imaging tools and models to reduce the impact of motion on the treatment. Online continuous nonionizing imaging has become possible with the recent development of magnetic resonance imaging devices combined with linear accelerators. This opens the way to new guided treatment methods based on the real‐time tracking of anatomical motion. In such devices, 2D fast MR‐images are well‐suited to capture and predict the real‐time motion of the tumor. To be used effectively in an adaptive radiotherapy, these MR images have to be combined with X‐ray images such as CT, which are necessary to compute the irradiation dose deposition. We therefore developed a method combining both image modalities to track the motion on MR images and reproduce the tracked motion on a sequence of 3DCT images in real‐time. It uses manually placed navigators to track organ interfaces in the image, making it possible to select anatomical object borders that are visible on both MRI and CT modalities and giving the operator precise control of the motion tracking quality. Precomputed deformation fields extracted from the 4DCT acquired in the planning phase are then used to deform existing 3DCT images to match the tracked object position, creating a new set of 3DCT images encompassing irregularities in the breathing pattern for the complete duration of the MRI acquisition. The final continuous reconstructed 4DCT image sequence reproduces the motion captured by the MRI sequence with high precision (difference below 2 mm).

## 1 INTRODUCTION

The goal of radiation therapy is to irradiate tumors while preserving healthy tissues and organs at risk. The treatment plan is generally designed on X‐ray 3DCT images to ensure conformity between the planned dose and its delivery. In the case of moving organs encompassing the respiratory movements, such as liver and lungs tumors, the treatment has to integrate the respiratory motion in a consistent way, from planning to delivery. One way to take this motion into account is to estimate it by using a 4DCT and provide motion margins to ensure the target coverage.^1^, ^2^, ^3^, ^4^ This lowers the risk of mistreating the target but also irradiates more healthy tissues, potentially creating complications.^5^ Unfortunately, a 4DCT is composed of reconstructed images, based on a couple of minutes of acquisitions sorted and resampled together into a sequence of 3D images. Therefore, it does not represent the real continuous motion but the average motion of one period computed over several breathing periods. Even if the 4DCT is acquired in the same position and with the same constraints as during the treatment, several studies have shown that during treatment, the motion induced by the breathing can differ significantly from the motion captured by the 4DCT in the treatment planning step,^6^ resulting in potential errors or suboptimal treatment.

Different methods have been developed to address the motion‐related issues and increase confidence in tumor localization. Gating based on an external surrogate can be used but relies on the basic assumption that the correlation between internal and external motion has been the same at planning and in the treatment room.^7^ Greater accuracy can be achieved by tracking fiducials implanted in the tumor,^8^ but this last solution requires heavy and risky invasive preintervention. Other methods have been developed to reduce the impact of breathing‐related motion during treatment such as abdominal compression,^9^ audio coaching,^10^ or mechanically assisted ventilation.^11^ These options yield lower motion amplitude or a more regular breathing pattern that can result in a first reduction of the motion margins, or a better gating or tracking precision.

The ultimate reduction in the motion margins would entail adapting the treatment in real‐time, based on precise tracking of the 3D anatomical structures’ volumes. This would make it possible to transform a complex 4D treatment into a sequence of more precise 3D treatments synchronized with the anatomical motion. To achieve this, the real time positions of the target and of the surrounding organs must be known throughout treatment delivery.

With the recent developments of hybrid Linac‐MRI solutions in standard photon based radiotherapy,^12^ continuous real time MR imaging during treatment is now possible and more research is focusing on online adaptive treatment^13^, ^14^, ^15^ and tumor tracking.^16^ In proton therapy, no hardware solution is as yet available, but is under serious consideration.^17^ MRI is the ideal imaging modality for this application. It can give good soft tissue contrast and it is not irradiant, which is crucial for an unconstrained use during treatment. Unfortunately, MRI devices do not allow real‐time full 3D acquisition for now and therefore only 2D slices can be acquired in real‐time during treatment to follow the motion (this is usually called cine or dynamic MRI). Full 3D motion must then be derived from this limited amount of geometric information using motion models^8^ Different methods have been developed to use 2D slices efficiently to drive 3D patient‐specific motion models built on vector fields coming from nonrigid deformation algorithms. The diaphragm position can be used as a navigator,^19^ a PCA‐based similarity metric can be derived from the vector fields,^20^, ^21^ or more recently ROI have been used to drive a motion model.^22^ All these options are used to transfer the 2D motion information from dynamic 2D MRI slices to complete 4D motion retrospectively. The treatment delivery quality can then be controlled between fractions and the accumulated dose for both the tumor and organs‐at‐risk can be computed, taking the patient's real breathing motion into account. However, these methods are not fast enough to be used in real‐time. Tumor tracking is sometimes possible using cine MRI 2D slices,^23^ but even with MRI soft tissue contrast the target is not always visible inside soft tissue such as in the liver.

While MRI is the ideal solution for continuous imaging during treatment, tissue density related images such as CT's are still necessary for dosimetric quantification. Recent results have shown that for standard radiotherapy and proton therapy alike, MRI‐only workflows can be precise enough to generate virtual 4DCT images derived from 4DMRI acquisitions. This method can replace 4DCT acquisition in the planning phase and be used for dose calculation and image guidance.^24^, ^25^

Another important issue is controllability. To be accepted as a real‐time treatment guiding tool, such a method has to give real‐time feedback to allow a treatment to be controlled and stopped in real‐time if necessary. To address these issues, we propose a method to transfer, in real time and continuously, the breathing‐induced anatomic motion tracked on 2D dynamic MRI to a virtual 4DCT sequence. Like the previously mentioned motion models, the proposed workflow is based on precomputed deformation fields, but the driving mechanism is a simple multimodal MRI‐CT interface tracking method. It is designed in such a way that is reliable and controllable through observation of the results in real‐time, allowing the practitioner in the treatment room to take immediate actions if necessary. It is also scalable in precision and can benefit from any future improvement in fast MR imaging. The method could be used as a treatment verification or guiding tool and as part of a real‐time dose accumulation observation method for photons or protons (see Discussion section).

## 2 METHOD

The initial step of our method relies on the acquisition or creation of a 4DCT (referenced as 4DCTo for original 4DCT) before treatment delivery. Prior to the real‐time application, deformation fields between the 4DCTo phases are computed using the diffeomorphic morphons algorithm^26^ from the open source platform OpenReggui.^27^ This algorithm computes 3D to 3D deformation fields that are consistent with the anatomy of the organs (diffeomorphism allows elimination of unrealistic artifacts in the deformation field) and can be summed and scaled easily while preserving rotations. Using the deformation from the morphons, we also compute the midposition (MidP) image^28^ offline prior to the real‐time application. Two deformation fields are saved for each phase N of the 4DCTo:

- The deformation field between the MidP phase and the phase N.
- The deformation field between the phase N and the phase N + 1 (the last phase is registered on the first one to create a cycle).

The continuous motion is captured through 2D dynamic MRI sequences. The acquisition frequency of the dynamic MRI is chosen high enough to be able to follow the breathing motion of the patient, creating a 2D video of the breathing pattern (See material section). The motion will be tracked on those image series using interfaces between moving anatomical structures.

**Fig. 1:**
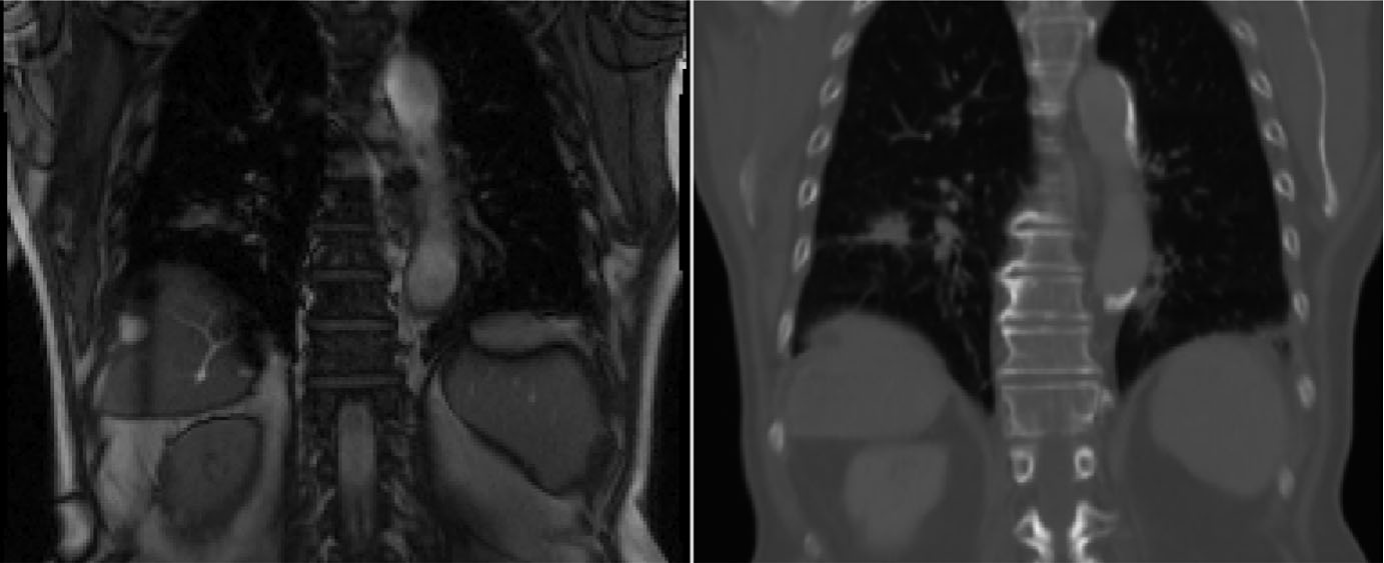
Example of 2DCT slice extraction after rigid registration of MRI on the 4DCTo.

While the 2D acquisition is running, the last step before launching the real‐time tracking is to find the position of the dynamic MRI plane inside the 4DCTo. This way, the corresponding 2DCT slices are extracted from the 4DCTo to form a short 2DCT video of 10 frames in the same plane position as the 2D MRI Fig. 1. This is done using the first few MRI frames of the sequence, based on the matching of nonmoving bony structures such as vertebrae. This can be done using multimodal rigid registration, but for the 15 patients in this study we did it manually. In a real treatment situation, it would be done right before the start of treatment.

**Fig. 2:**
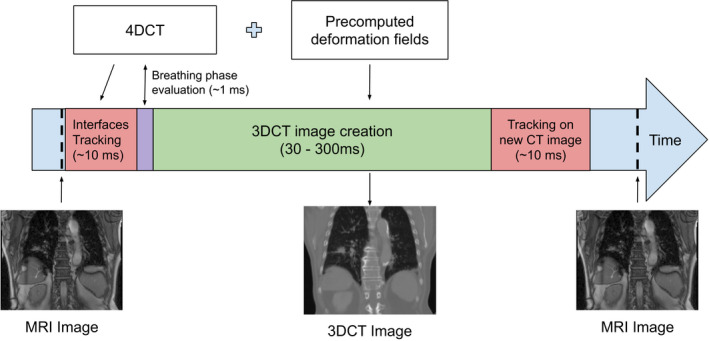
3D real‐time motion reproduction workflow.

The general workflow of our approach is summarized in Fig. 2. Using the two data sets, namely the continuous 2D MRI and the 4DCTo with the associated deformation fields, our method transfers the positions of tracked anatomical structures from the 2D MRI frames to a new set of virtual 3DCT phases in real‐time. A 3DCT image is generated for each MRI frame by inter‐ or extrapolating the deformation fields to constitute the continuous virtual 4DCT (4DCTc). Note that by using precomputed deformation fields to deform 3DCT images, our method relies on the hypothesis that breathing‐unrelated anatomical changes such as stomach fullness, bowel gas, tumor growth/shrinkage and patient weight loss stay small between the 4D image set acquisition and the application of our method, at least in the treatment path. Under this condition, the entire 3D motion description based on a small set of 2D slices is reliable. If it is not the case, the quality of our results might drop but the validation step which is also part of the real‐time workflow will detect the difference and let the user to take action if necessary (see discussion). The different steps in the process are detailed in the following subsections.

### 2.A Interface tracking

**Fig. 3:**
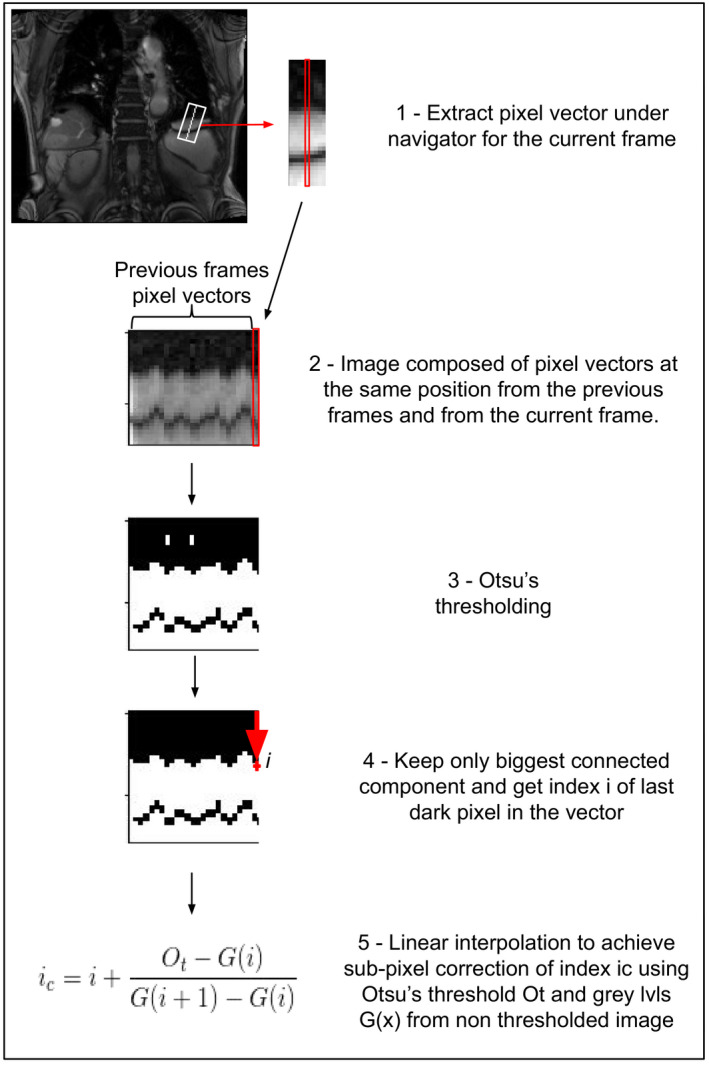
Steps of the interface tracking method.

The breathing‐induced organ motion and positions are captured by tracking the interfaces between two tissues. To track an interface, the user can place a navigator, a small line in the direction of the motion and crossing the interface to track. The tracking works with a simple image processing pipeline, Fig. 3 occurs in real‐time (~2 ms per navigator) and is robust to noise. The name “navigator” is borrowed from the MRI navigator echoes, a 1D acquisition MRI scanners can acquire and use to sort images in post processing or trigger acquisitions.^29^ ^,^ ^30^ In this case, the 1D vector is a vector of pixels manually selected on the frame, but the outcome is similar. We chose to position them manually for reasons linked to reliability and controllability by the practitioner and are developed in the discussion section.

**Fig. 4:**
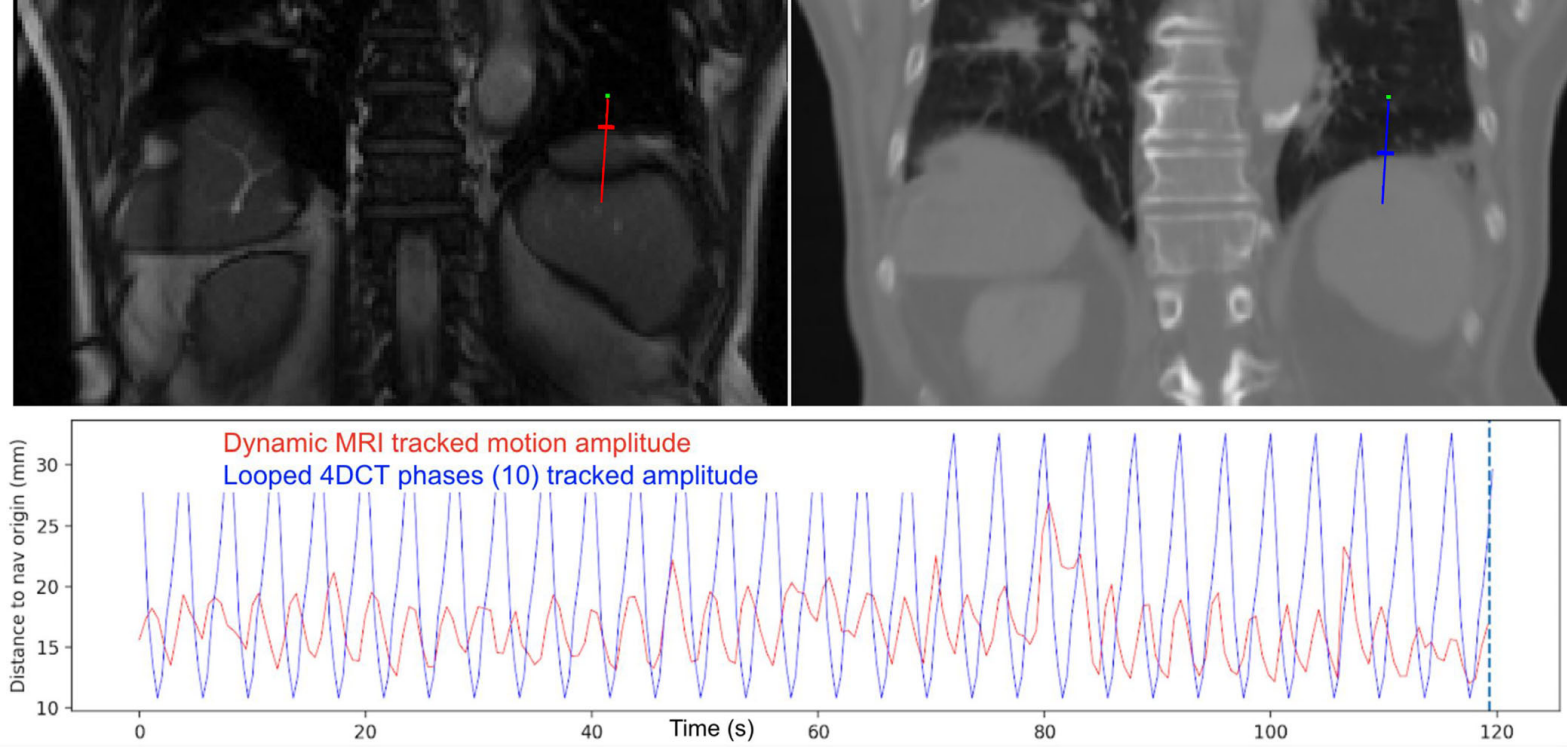
Example of motion amplitude tracked on both the 216 frames of the dynamic MRI (in red) and on the 10 looped frames of the 4DCTo (in blue). The tracked motion is given as the distance between the navigator end (green dot) and the tracked interface (small perpendicular line) in mm.

When an interface is chosen on one image series, a navigator pair is automatically created to track the same interface on both image series using the registration of the MRI position on the 4DCTo. The tracked position is given as the distance between the navigator end and the tracked interface in mm. The interface motion measured on the MRI frames is compared to the one measured on the 10 frames of the 2DCT video Fig. 4.

As the aim is to reproduce with fidelity the motion of the tumor, of surrounding tissues and of anatomical structures in the path of a photon or proton beam, these regions are our main concern and were chosen in priority on all the sequences to place the navigators. A set of two to five navigator pairs was used for each MRI slice position, depending on the quantity of interesting tissue interfaces that were visible on both the MRI and CT images. In most cases, tumor borders were not visible enough to be tracked on either the MRI or CT image series. We chose interfaces with which the tumor motion seemed highly correlated, but we did not compute the correlation coefficient for this work.

### 2.B Phase selection method

Once the interface position is measured on the 10 phases of the 4DCTo and at the same position for the new MRI slice, both motion signals are combined to determine which phase has to be created with the following steps:

1 Check the current breathing state.

**Fig. 5:**
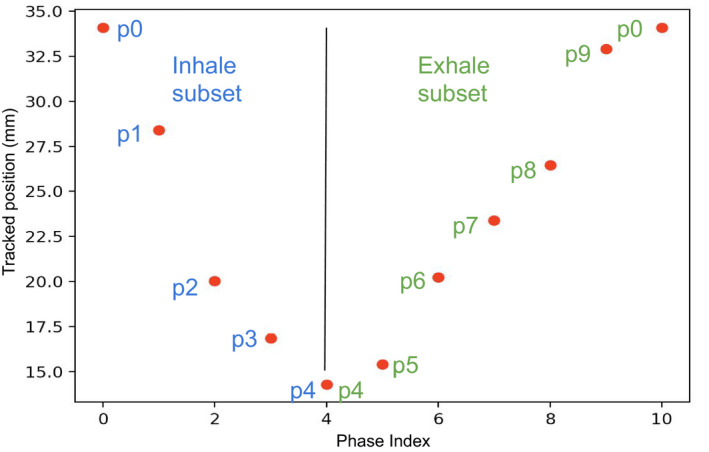
Separation of the 2 phase subsets.

Comparing the positions measured between the new MRI frame and the previous one determines the current breathing state as inhaling or exhaling. The state is then used to select the corresponding subset in the 4DCTo phases [Fig. 5].

2 Find the closest phase in the breathing state subset of CT phases.

The breathing amplitude position measured on the MRI frame is compared with the amplitudes of the selected subset of CT phases to find the closest phase from the MRI amplitude.

3. Compute an inter‐ or extrapolation ratio by comparing the measured motions.

**Fig. 6:**
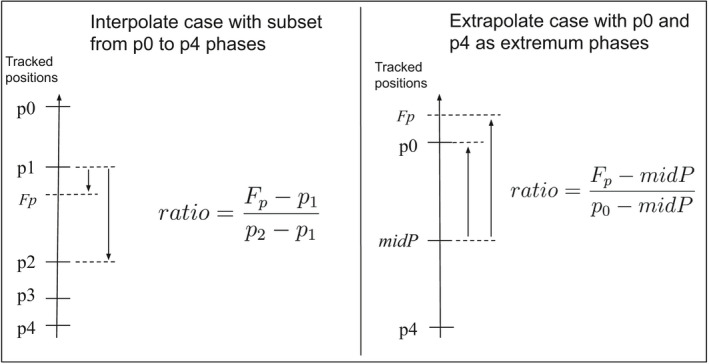
Example of ratio computation in the case of interpolation (left) and extrapolation (right). *Fp* is the new frame position and *midP* is the mean position of all the phases (not just the subset).

A ratio index is computed using the positions measured on the MRI frame and on the closest CT phase found in Point 2 [Fig. 6]. Two cases must be considered:

‐ Interpolation: The MRI position *Fp* is inside the range of the CT phases amplitude
‐ Extrapolation: The MRI position *Fp* is outside the range of the CT phases amplitude

**Fig. 7:**
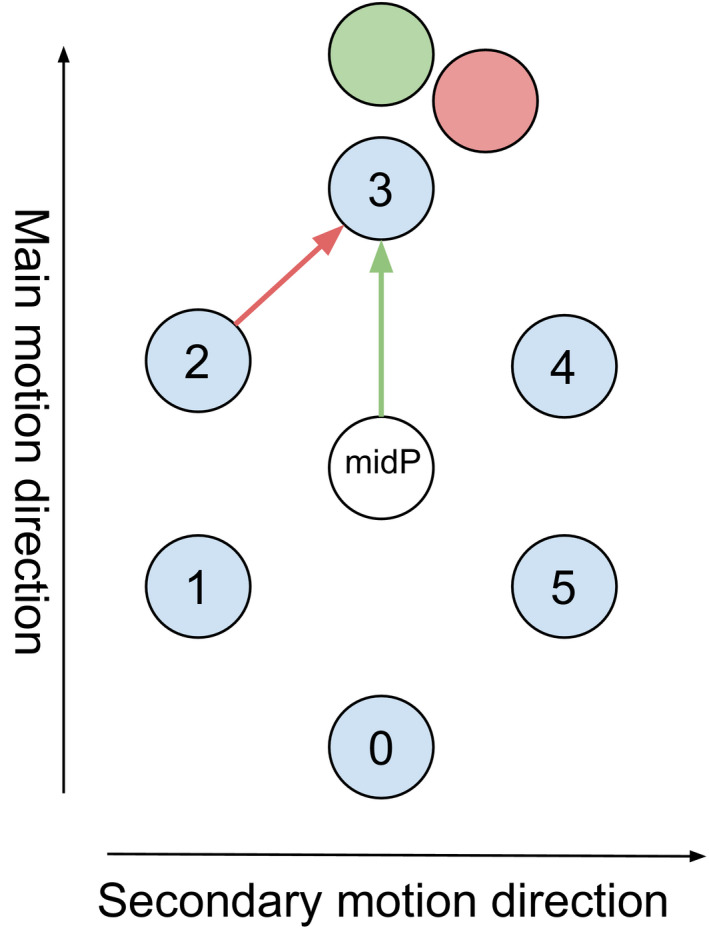
Example of hysteresis trajectory of an object in 2D. Positions in the 4DCTo phases are represented in blue. Results of extrapolation of the deformation fields are shown using the midP phase (green) or the N‐1 phase (red). The motion in the second direction is highly exaggerated for illustration purposes but shows the difference that the two approaches can create.

For the extrapolation case, we decided to use the MidP phase to compute the ratio to avoid bending the motion trajectory away from the main motion direction in the case of hysteresis motion as represented in Fig. 7.

### 2.C Multinavigator average phase selection

**Fig. 8:**
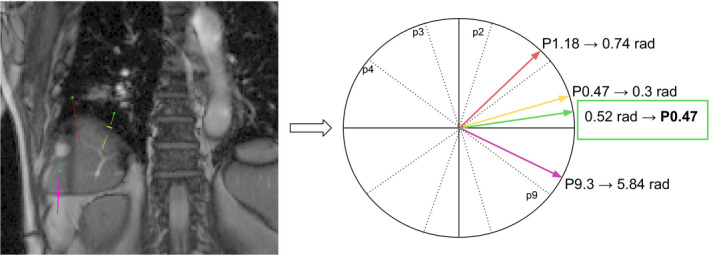
Example of average vector and resulting phase (in green) between the three navigators after transformation of phases *P* into angles and vectors.

To reduce image noise impact on the phase selection method, the resulting phase estimations coming from the *n* navigators are combined to compute an average of the resulting phases Fig. 8. First, the phases $p_{n}$ are transformed into angles $\theta_{n}$ between 0 and 2π using the number of phases *N* in the 4DCTo Eq. (1), such that the phases become periodic and phase 0 follows phase 9. In this set of angles, if a phase given by a navigator is too far from the others, it is considered an outlier and removed. Then, the remaining angles are used to compute a new angle $\theta_{f}$ corresponding to the average of sines and cosines of angles $\theta_{n}$ Eq. (2). Finally, the angle $\theta_{f}$ is transformed back into the final phase value $p_{f}$ Eq. (3).

(1) $$\begin{array}{c} \theta_{n}=\frac{2\pi p_{n}}{N}\# \end{array}$$

(2) $$\begin{array}{c} \theta_{f}\equiv \left\{\begin{array}{c} \cos\theta_{f}=\frac{1}{n}\sum_{n}\cos\theta_{n}\\ \sin\theta_{f}=\frac{1}{n}\sum_{n}\sin\theta_{n} \end{array}\right)\# \end{array}$$

(3) $$\begin{array}{c} p_{f}=\frac{N\theta_{f}}{2\pi }\# \end{array}$$

### 2.D 3DCT phase creation

The final step is to generate the new 3DCT phase. The velocity field between two phases, N and N + 1 in interpolation cases or MidP and N in extrapolation cases, is multiplied by the interpolation or extrapolation ratio. Then, in order to use it to deform an image, the velocity field has to be converted into a deformation field using field exponentiation. Finally, the phase N is deformed using this interpolated or extrapolated deformation field to generate a new 3D phase matching the MRI motion amplitude.

We ran this method on a decent laptop. Creating the entire 3DCT image for each MRI frame took around 2 sec by frame. It is too much to be used in real time, since we only have from 220 ms to 320 ms between two frames (See Materials). However, limiting the region to deform to the areas around the navigators and the treatment path greatly reduces the time required to create the image. To determine this area of interest, we used the treatment plan to get the treatment path, on which we added 2 cm on every side before cropping the image. The creation of such a partial image took only between 30 and 300 ms. This can still be improved by using dedicated hardware (see Discussions). Full images were also created anyway (not in real‐time), for the sake of simplicity for displaying and observing the results.

### 2.E Validation

The evaluation of the quality of the results is also part of the real‐time process. To see if the motion of the MRI is well‐matched, pairs of navigators are applied again, this time on the current MRI frame and on the 3DCT we have created for this frame. As the MRI contains the ground truth anatomy that we aim to replicate with the 4DCTc, this difference should be as small as possible. This comparison is done for two navigator sets:

‐ Used navigators set (UN): Navigator positions that were used previously and from which the new image has resulted.
‐ New navigators set (NN): Navigator positions that were not used previously to create the new image.

The absolute value of the difference between the two positions is computed for every new frame and all navigator pairs. At the end of the sequence, the average of this difference along the entire 4DCTc is computed.

The difference is measured for multiple navigators, but all in the same slice. That means that the farther we go from the MRI slice position, the less likely our deformed image is to match the patient's real anatomy exactly. If the tracking difference is low even for navigators of the NN set placed far away from the rest of navigators (more than 20 cm away, for example), it shows that there was not a lot of nonbreathing‐related changes between the two images and that our hypothesis is verified. It is a good indication that the deformation in the third dimension should also behave well. If a larger difference is measured between the tracking of a navigator pair, it signals that either that nonbreathing‐related anatomical changes occurred or that the breathing pattern deviates greatly from what is contained in the 4DCTo (a big extrapolated case, for example).

The important part of the image is the beam path and we think a small set of 2D slices is enough to capture with precision what happens in this area, with the possibility of linking the treatment to the acquisition so as always to acquire images where the beam is currently irradiating.

Interleaved planes or multiple slices in the same plane, while not technically a 3D validation, are used to validate the quality of the transferred motion for different planes successively, several times each second.

### 2.F Materials

Patient data come from 15 patients treated for primary (hepatocarcinoma) or secondary (metastasis) liver tumors by radiotherapy. For their treatment, a 4DCT was acquired in the treatment position, with abdominal compression and audio‐coaching to regularize the breathing pattern. We also have treatment plans for these patients. We did not use them to compute doses but simply to get the treatment path (See 3DCT phase creation). This trial was carried out in accordance with The Code of Ethics of the World Medical Association (Declaration of Helsinki) for experiments involving humans, approved by our local ethics committee (B403201628906). Informed consent was obtained from all participants before the trial.

The MRI sequences were True FISP sequences from a Siemens Skyra 3T scanner. These were acquired the same day and under the same conditions as the 4DCT, with abdominal compression and audio‐coaching. We acquired one to four different sequences on each patient using different numbers of slice positions, slice orientations, and acquisition frequencies. Some of the sequences were acquired several times, depending on the patient's comfort during the MRI or the available scanner time. The acquisition frequency was set to between 1.5 Hz and 3.16 Hz so that we could follow the breathing motion while keeping pixel size under 3 mm for image quality (see discussion). The different slice position choices and acquisition parameters are described below.

1. Single slice in sagittal or coronal plane (14 sequences for 14 positions)
  ‐ 3.16 Hz for 380 slices in 2 min
  ‐ TE: 1.41, TR: 3.23, 208 × 204 pixels of 1.6 × 1.6 × 7 mm
2. 2 interleaved coronal and sagittal slices (12 sequences for 24 positions)
  ‐ 1.83 Hz for both orientation (3.66 Hz in total), 440 slices (2 × 220) in 2 min
  ‐ TE: 1.39, TR: 3.16, 188 × 192 pixels of 1.82 × 1.82 × 7 mm
3. 3 contiguous slices in sagittal or coronal plane (12 sequences for 36 positions)
  ‐ 1.5 Hz for the 3 slices (4.5 Hz in total), 540 slices (3 × 180) in 2 min
  ‐ TE: 1.33, TR: 3.01, 126 × 128 pixels of 2.5 × 2.5 × 7 mm

In total, 74 different 2D slice positions acquired for 2 min were available to track the breathing motion for a total of more than 17000 images. Every position was used independently, meaning that when the two interleaved coronal and sagittal slices were used, each frame of each orientation was used to track the motion and to create a 3DCT image.

## 3 RESULTS

For partial images limited to the navigators and the beam path areas, it takes 2 ms per navigator to track borders positions, between 30 and 300 ms to create the new deformed image and 2 ms per navigator to track the borders again on the new image to evaluate its quality. The complete process takes between 40 and 320 ms depending on the number of navigators and the size of the area to deform.

**Fig. 9:**
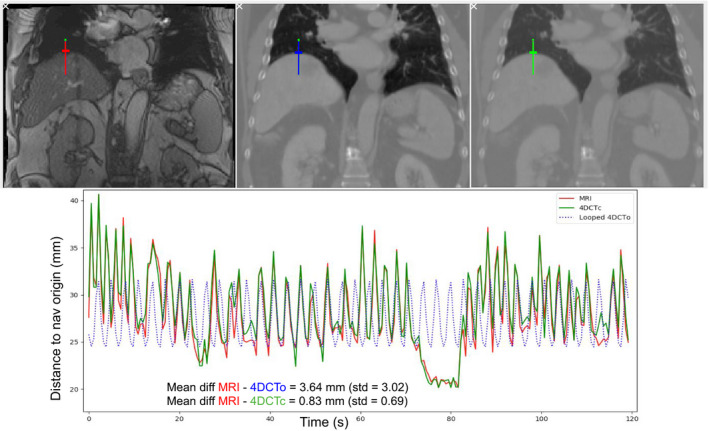
Example of motion comparison between the MRI sequence, the created 4DCTc and the looped 4DCTo and the resulting mean difference between motion signals.

**Fig. 10:**
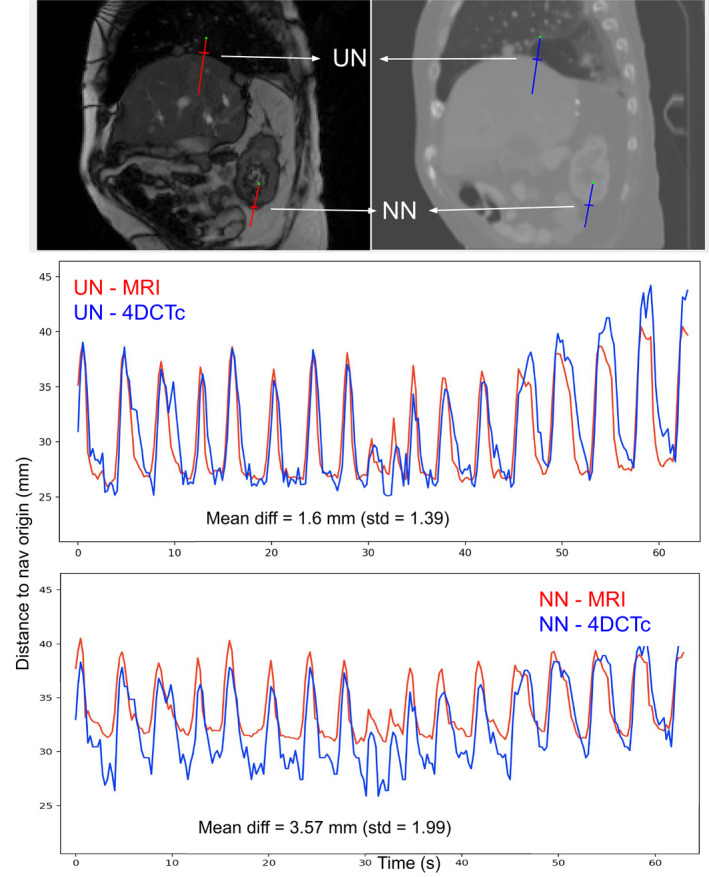
Example of motion comparison between MRI and 4DCTc in UN (top) and NN (bottom) cases.

When measured on the navigators of the UN set, the absolute value of the motion signals difference between the MRI and the 4DCTc was usually under 2 mm in average during the 2 min of acquisition (Example in Fig 9). The average and maximum difference for all patients are reported in Table 1. The same results for the NN navigator set are reported in Table 2 and the difference between the MRI and the 4DCTc motion was for most sequences higher than in the UN cases. We observed that even if the frequency of the motion was well‐matched, the amplitude of the MRI was not always reproduced correctly and a shift between both motion signals could appear Fig 10, especially for navigators of the NN set if placed far from the UN ones. Note that the max difference corresponds to the worst case of all our images and for all our navigators (more than 17000 images with several tracked positions on every image).

**Table 1:** Results for motion measured at the phase selection positions (UN) for all patients, separated by pixel spacing (1.6, 1.82, or 2.5 mm).

UN cases	Mean diff 4DCTc	Max diff 4DCTc	Mean diff 4DCTo	Max diff 4DCTo
1.6 mm (14 positions)	1.75 mm (std = 0.76)	12.06 mm	9.4 mm (std = 6.2)	33 mm
1.82 mm (24 positions)	1.64 mm (std = 0.9)	11.34 mm	8.94 mm (std = 7.1)	37 mm
2.5 mm (36 positions)	2.03 mm (std = 1.4)	12.22 mm	7.34 mm (std = 2.23)	29 mm

**Table 2:** Results for motion measured at other positions (NN) for all patients, separated by pixel spacing (1.6, 1.82, or 2.5 mm)

NN cases	Mean diff 4DCTc	Max diff 4DCTc	Mean diff 4DCTo	Max diff 4DCTo
1.6 mm (14 positions)	2.48 mm (std = 0.6)	9.58 mm	9.14 mm (std = 5.45)	24 mm
1.82 mm (24 positions)	2.2 mm (std = 1.18)	19.57 mm	6.08 mm (std = 2.52)	32.92 mm
2.5 mm (36 positions)	3.92 mm (std = 2.22)	11.3 mm	7.97 mm (std = 2.77)	31 mm

The same results are reported in Table 1 and 2 for the difference between the MRI and the 4DCTo where the looped 4DCTo was synchronized with the audio coaching loop. It is reported as a comparative element and shows the gain of copying the motion using our method vs simply using the 4DCTo.

**Fig. 11:**
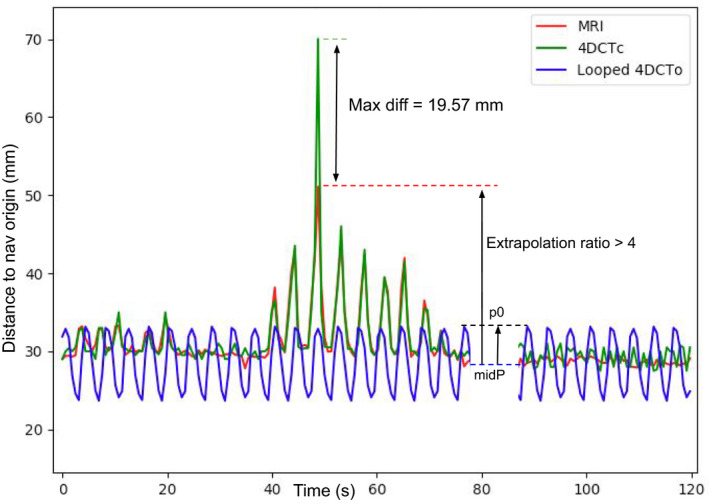
Example of extreme extrapolation case multiplying small errors.

The MRI motion was usually well‐reproduced by the 4DCTc, even with irregular breathing patterns Fig 8. However, our method's precision started to drop in extreme cases of motion extrapolation, where the MRI tracked point was really far out of the 4DCTo range. This is caused by the fact that extrapolation also multiplies the errors by the extrapolation phase ratio. In some extreme cases, it can create a big difference between the MRI frame amplitude and the reconstructed 4DCTc phase Fig 11. Of all the 74 MRI positions used, the worst results shown in Table 1 and Table 2 by the maximum difference column were all results coming from extrapolation cases with high extrapolation ratios.

The image resolution did not seem to have an important impact on the motion tracking and reproduction quality. But to be as close as possible to the real anatomy of the day, we would recommend a resolution as high as possible to represent the tracked interfaces’ positions more precisely and ensure a higher quality 4DCTc.

## 4 DISCUSSION

### 4.A 4DCT reliability and interfraction motions

As mentioned in the method section, we suppose that interfraction variations such as tumor baseline shift, tumor shrinking, and stomach/bladder filling will be checked prior to delivering the daily fraction, using online pretreatment MRI imaging, for example. Parts of the anatomy in the 4DCTo can be deformed or shifted compared with continuous MRI and the slightly worse outcome of our measurements in NN cases compared with UN cases confirms that our hypothesis was not always verified everywhere in the anatomy. The validation step applied as part of the real‐time workflow is there to verify the hypothesis during the entire application and can be used to raise a flag and stop the treatment if necessary.

Generally speaking, the more recent the image the more reliable it is. For this reason, updating the 4DCTo and corresponding deformation fields right before every fraction would be ideal. Options are being developed using, for example, only one 3DCT image and a 4DMRI.^31^ This way, it might be possible to generate a new 4DCT several times over the treatment period, each time an irregular change happens, without increasing the imaging dose given to the patient too much. This could be a complement to the setup phase of today's practice, in which images are already acquired to position the patient. If done in the treatment room, such daily acquisition would probably increase the length and cost of the treatment session, but if the motion margins can be removed with a new improved guided treatment, it might be worth it.

As our next step, we are working on a way to apply this method even in the case of interfraction changes between the anatomy of the day and the 4DCTo. In any event, it is important to focus on motion in the beam's path to create the 4DCTc to get the best results for a treatment verification/guiding tool.

### 4.B Manual tracker positioning

We chose to use a manual tracker positioning for different reasons. First, it lets one select an interface visible on both MRI and CT modalities, which is necessary for our method. Then, it allows to avoid MRI artifacts (balanced steady‐state banding artifacts^32^) and 4DCT reconstruction artifacts. Finally, it allows the operator to select a moving interface in the path of the treatment and with which the target's motion is correlated. That is important because in the liver, the target is not often visible on images and it may not be possible to use the target as a tracking interface. The choice of correlated motion is important and must be done carefully, because 20% of liver tumors have been shown to follow a hysteresis trajectory and the liver can deform nonrigidly during the breathing cycle.^33^ If the target is clearly visible on the MR images, it is usually not difficult to either find a correlated motion or at least check if the target's motion deviates too much from the motion used to create the CT images.

### 4.C Image acquisition frequency vs resolution and real‐time use

In MRI, there is always a trade‐off between acquisition frequency and image resolution. For this application, the priority was to follow the breathing motion in real time, including irregularities such as coughing or swallowing. For this, we recommend having at least 10 images by breathing period. Some patients can have a really fast breathing pattern, with a period under 3 sec. In these cases, the image resolution and resulting precision of the tracking could be affected.

In the case of a fast breathing pattern, it might also be necessary to predict the motion to account for the image acquisition and image processing time. Motion amplitude prediction using the previous frames and the expected breathing parameters such as period and amplitude could be used to anticipate better which 3DCT to use. Note that this is not ideal because the point of a real time tracking is not to depend on predictions. If such a predictor is necessary, each prediction would need to be validated using the following frame to avoid accumulating errors.

We did not mention the latency between the moment a motion occurs and the moment it appears in the MR images due to the imaging time image reconstruction time, and data transfers. It must be considered to implement a clinical real‐time application. In the best case, it has been reported to be around 150 ms^34^ for fast acquisitions on an MR‐Linac. With MR‐Linacs now available and online treatment guidance in development, we can hope for improvements in image acquisition and reconstruction speed, as well as in data transfer time.

Also, between MR slices, navigator echoes could be used to improve the temporal resolution of the tracking, as long as they can be positioned on an interface visible on the navigator echo specific acquisition and CT images. Studies have shown that it can be used to track interfaces on top of dynamic MRI images.^35^ They have the advantage of being fast to acquire and process.

For a real‐time use, several applications have to be considered:

- In order to be used as an offline tool to recompute the fraction dose afterwards, only the MRI acquisition needs to be fast to capture any irregularities in the breathing pattern. The method presented here can be used offline.
- Using this tool as a real‐time anatomy‐based guiding tool with preplanned treatment means that tissue interface tracking and breathing state evaluation have to be done in real time, but these steps take only a few milliseconds. The main issue is the MRI acquisition and reconstruction time.
- To be used as a real‐time dose‐based verification or guiding tool and to stop the treatment if some criterion about the given dose is reached, the dose must be computed continuously and accumulated before any error in dose delivery becomes too great. In this case, the 3DCT image has to be created using deformation as part of the real time application. On this deformed image, the dose delivered during the current image timeframe must be computed as fast as possible to be able to raise a flag if necessary, before too much dose is given to healthy tissues. A dose computation method with such speed is difficult to achieve, but fast methods already exists^36^, ^37^ and may become even faster in the future.

## 5 CONCLUSION

The proposed method can generate in real time a set of 3DCT images that imitates well the motion observed on dynamic MRI. Our method relies on a simple real‐time interface tracking method and on precomputed 3D deformation fields. It could be used to recompute the delivered dose between treatment fractions more precisely, and eventually trigger a plan adaptation. It could also be used to compute the delivered dose in real time, either as a real‐time verification tool or in the future as a real‐time guiding tool for an on‐the‐fly adapted treatment method. Positioning the trackers manually requires a human intervention, but it also makes the method more robust to imaging artifacts or noise and gives the physician final control. The resulting 4DCTc quality is verified in real‐time using the same tracking tool. Our next step will be to use it to compare different treatment strategies to reduce the motion margins. The different treatment strategies outcomes can be compared by recomputing the delivered dose on the 4DCTc with consideration of the real motion tracked on the MRI sequences.

## CONFLICT OF INTEREST

Kevin Souris is now involved in a research collaboration with IBA s.a. and is funded by the Walloon Region (Belgium).
